# PDGFRβ + cell HIF2α is dispensable for white adipose tissue metabolic remodeling and hepatic lipid accumulation in obese mice

**DOI:** 10.1186/s12944-024-02069-1

**Published:** 2024-03-20

**Authors:** Tao Yao, Danni Wei, Xin Tian, Lin Zhao, Qiangyou Wan, Xiaoli Zhang, Juan Cai, Siqi Li, Bowen Diao, Suihan Feng, Bo Shan, Mengle Shao, Ying Wu

**Affiliations:** 1 College of Life Sciences, Zhejiang Chinese Medical University, Hangzhou, China; 2 Zhejiang Academy of Traditional Chinese Medicine, Tongde Hospital of Zhejiang Province, Hangzhou, China; 3 Key Laboratory of Immune Response and Immunotherapy, Shanghai Institute of Immunity and Infection, Chinese Academy of Sciences, Shanghai, China; 4 University of Chinese Academy of Sciences, Beijing, China; 5 Cancer Center, Zhejiang University, Hangzhou, China; 6 Zhejiang Provincial Key Laboratory of Pancreatic Disease, The First Affiliated Hospital, Institute of Translational Medicine, Zhejiang University School of Medicine, Zhejiang University, Hangzhou, China

**Keywords:** Hypoxia-inducible factor, Adipose tissue, Adipocyte progenitor, Obesity, Hepatic steatosis

## Abstract

**Background:**

Obesity is associated with extensive white adipose tissue (WAT) expansion and remodeling. Healthy WAT expansion contributes to the maintenance of energy balance in the liver, thereby ameliorating obesity-related hepatic steatosis. Tissue-resident mesenchymal stromal cell populations, including PDGFRβ + perivascular cells, are increasingly recognized pivotal as determinants of the manner in which WAT expands. However, the full array of regulatory factors controlling WAT stromal cell functions remains to be fully elucidated. Hypoxia-inducible factors (HIFs) are critical regulators in WAT stromal cell populations such as adipocyte precursor cells (APCs). It is revealed that HIF1α activation within PDGFRβ + stromal cells results in the suppression of de novo adipogenesis and the promotion of a pro-fibrogenic cellular program in obese animals. However, the role of HIF2α in PDGFRβ + cells remains undetermined in vivo.

**Methods:**

New genetic models were employed in which HIF1α (encoded by the *Hif1a* gene) and HIF2α (encoded by the *Epas1* gene) are selectively inactivated in PDGFRβ + cells in an inducible manner using tamoxifen (TAM). With these models, both in vitro and in vivo functional analysis of PDGFRβ + cells lacking HIF proteins were performed. Additionally, comprehensive metabolic phenotyping in diet-induced mouse models were performed to investigate the roles of PDGFRβ + cell HIF proteins in WAT remodeling, liver energy balance and systemic metabolism.

**Results:**

Unlike HIF1α inactivation, the new findings in this study suggest that inducible ablation of HIF2α in PDGFRβ + cells does not cause apparent effects on WAT expansion induced by obesogenic diet. The adipogenic ability of PDGFRβ + APCs is not significantly altered by genetic HIF2α ablation. Moreover, no difference of key parameters associated with healthy WAT remodeling such as improvements of WAT insulin sensitivity, reduction in metabolic inflammation, as well as changes in liver fat accumulation or systemic glucose metabolism, is detected in PDGFRβ + cell *Epas1*-deficient mice.

**Conclusion:**

The new findings in this study support that, in contrast to HIF1α, PDGFRβ + cell HIF2α appears dispensable for WAT metabolic remodeling and the resulting effects on liver metabolic homeostasis in diet-induced obesity, underscoring the isoform-specific roles of HIFα proteins in the regulation of adipose tissue biology.

**Supplementary Information:**

The online version contains supplementary material available at 10.1186/s12944-024-02069-1.

## Introduction

Obesity is associated with extensive WAT expansion and complex WAT metabolic remodeling [1, 2]. The prevailing notion proposes that factors beyond mere increased adiposity drive metabolic impairments associated with obesity [3, 4]. Nonalcoholic fatty liver disease (NAFLD) occurs when an excess amount of fat builds up in the liver. Genetic factors confer susceptibility to NAFLD, with missense variants in patatin-like phospholipase domain containing protein 3 (PNPLA3) and in transmemebrane 6 superfamily member 2 (TM6SF2) identified as 2 strongest genetic risk factors [5–8]. The onset and development of NAFLD are also influenced by other risk factors such as obesity [9]. Its progression into nonalcoholic steatohepatitis (NASH) involves liver inflammation and damage, along with fat accumulation. NAFLD and its most severe form NASH are swiftly emerging as the primary cause of chronic liver conditions [10, 11], propelled by the rising global prevalence of obesity [9, 12]. However, there are no approved pharmacological treatments for NAFLD and NASH. Given the complicated nature of its pathophysiology, different pathways are under investigation for potential therapeutic strategies, with the focus on mitigating inflammation, resolving fibrosis and promoting metabolic health in obesity [13].

An emerging determinant of metabolic health in obesity is the mode by which WAT expands and remodels [1, 14, 15]. In principle, WAT expansion can occur through the enlargement of cell size (adipocyte hypertrophy) or the increase in cell number (adipocyte hyperplasia) [16]. Hypertrophic WAT expansion is a hallmark of pathologic obesity which is featured by WAT inflammation and fibrosis, adipocyte dysfunction, ectopic lipid accumulation and early onset of insulin resistance [17]. On the contrary, hyperplastic WAT expansion is metabolically protective as individuals with smaller and more numerous adipocytes within WAT often have lower levels of WAT inflammation and fibrosis, along with preserved insulin sensitivity [18, 19]. Of note, obese individuals with preferential subcutaneous fat accumulation are relatively resistant to the onset of metabolic disorders such as insulin resistance, dyslipidemia and hepatic steatosis [20, 21]. When overwhelmed by excessive energy intake, adipose tissue expands to its maximum capacity, leading to the overflow of surplus lipids into the circulation [22]. Elevated circulating lipids reach the liver, promoting hepatic fat accumulation. Moreover, dysregulated adipokine secretion along with pro-inflammatory factors from dysfunctional adipose tissue further exacerbate liver damage, eventually resulting in heightened hepatic lipid accumulation [14]. Furthermore, adipose tissue dysfunction triggers the development of insulin resistance in non-adipose organs/tissue, including the liver, contributing significantly to systemic metabolic disturbances that drive fatty liver development [14, 23].

Previous study revealed that increased recruitment of new adipocytes from de novo adipogenesis from specialized progenitors can prevent pathological remodeling and preserve WAT metabolic health [24]. Enhanced adipogenic differentiation of *Pdgfrb*-expressing adipocyte precursors through overexpression of *Pparg* (the master adipogenic transcription factor) prevents pathologic WAT expansion, improving hepatic fat steatosis and systemic glucose homeostasis in diet-induced obesity [24]. Thus, targeting adipocyte progenitor differentiation holds promise for maintaining liver metabolic health in obesity.

The emergence of single-cell technology has greatly powered the phenotypic discoveries at unprecedented resolution and the revelation of adipocyte progenitor functional heterogeneity [25]. Of particular interest has been the WAT depots in which a well characterized array of cell types coordinates to shape the microenvironment and determine the manner in which WAT remodels in obesity [26–35]. The recent identification of PDGFRβ + mesenchymal progenitor subpopulations has exemplified the remarkable functional differences between molecularly distinct adipocyte progenitor cells [27, 36–38]. Besides defining the distinctive functional properties of progenitor subpopulations using single cell RNA sequencing (scRNA-seq), comprehensive transcriptomics and quantitative proteomics have uncovered regulatory mechanisms which functionally distinguish adipose progenitor subpopulations and determine the cell fates of these cells [39].

Of note, HIF signaling pathway has been identified as a key determinant of adipose progenitor function [40, 41]. HIF proteins belong to the basis helic-loop-helix Per-Arnt-Sim (bHLH-PAS) transcription factor superfamily. HIF proteins function as heterodimers, comprising an oxygen-sensitive α-subunit (HIF1α, HIF2α or HIF3α) and a constitutively expressed β-subunit (HIF1β) [40, 42]. HIFα proteins are unstable and subjected to ubiquitin-mediated proteolysis in normoxic conditions [43]. During hypoxia, HIFα subunits are rapidly stabilized and activates HIF target gene expression [43]. In the context of pathologic obesity, grossly expanding WAT outpaces inadequate neovascularization, and leads to compromised oxygen supply and a higher degree of hypoxia in local micromilieu [14, 15]. This unfavorable microenvironment exacerbates insulin resistance, impairs metabolic regulation, and fosters a pro-inflammatory phenotypic program within WAT, which eventually contributing to systemic complications like cardiovascular disease, diabetes and liver disease [15, 44]. Thus, WAT hypoxia in obesity represents a pivotal factor in the pathophysiology linking excess adiposity to metabolic dysregulation and associated comorbidities.

Notably, WAT hypoxia is considered as a key regulatory factor contributing to extracellular matrix (ECM) remodeling and ECM stress [45–47], and local inflammation through HIF-dependent mechanisms, in particular HIF1α signaling activation [45–47]. Suppression of adipocyte HIF1α activity leads to metabolic improvements in obese animals [48–52]. Alternatively, metabolically stressed cells with elevated cellular oxygen consumption can experience a state of “pseudo-hypoxia” which leads to HIF protein activation [53]. In mature white adipocytes, oxygen consumption-induced HIF1α activation is an initiating trigger for WAT inflammation and dysfunction [49, 53]. Notably, HIF2α inactivation in mature adipocytes does not share same phenotypic consequences with HIF1α ablation [49]. In contrast, adipocyte HIF2α appears to confer protective effects in a number of contexts [54–56].

In addition to the roles in mature adipocytes, the contribution of HIF proteins in non-parenchymal cells to obesity-associated WAT remodeling has also been documented. For instance, genetic Hif1α ablation in myeloid cells protects against the development of HFD-induced metabolic inflammation and insulin resistance [57–59], while macrophage HIF2α suppresses inflammation and alleviates obesity-related insulin resistance [60, 61]. These studies collectively underscore the unique activities of HIFα isoforms in the regulation of WAT physiology and metabolic health.

Shao, M. et al. recently revealed that HIF1α-induced PDGFRβ signaling drives inhibitory PPARg phosphorylation and triggers pathologic WAT expansion in obesity [36]. Pharmacological and genetic suppression of HIF1α activity promotes PDGFRβ + progenitor adipogenesis and improves WAT metabolic function [36]. scRNA-seq transcriptomic data revealed the differential expression of HIF protein target genes in WAT resident PDGFRβ + cell subpopulations [27, 36]. Both HIF1α and HIF2α can be effectively activated by chemical stabilizers in PDGFRβ + cells isolated from WAT [36]. However, the role of HIF2α in these cells remains to be fully described.

Here, a TAM-inducible model of PDGFRβ + cell specific HIF2α inactivation was utilized to determine the regulatory role of HIF2α in controlling adipose progenitor function and WAT metabolic remodeling in the context of diet-induced obesity. The results revealed that HIF2α ablation in PDGFRβ + cells does not remarkably impact PDGFRβ + cell function. The frequency and adipogenic ability of PDGFRβ + cell subpopulations are not significantly affected by HIF2α inactivation. In agreement, changes of high fat diet (HFD)-induced WAT metabolic remodeling are not detected when HIF2α is inactivated in PDGFRβ + cells. The lack of phenotype in WAT in PDGFRβ + cell *Epas1*-deficient mice is consistent with similar levels of key metabolic parameters reflective of hepatic and systemic metabolic health, in contrast to PDGFRβ + cell *Hif1a*-deficient obese mice. Collectively, the data in this study support that PDGFRβ + cell HIF2α is dispensable for WAT metabolic remodeling and the consequential effects on liver and systemic metabolic homeostasis in diet-induced obesity.

## Materials and methods

### Animals and diets

*Pdgfrb*-CreER^T2^ [B6.Cg-Tg(*Pdgfrb*-cre/ERT2)6096Rha/J, JAX#:029684] strain was acquired from The Jackson Laboratory. *Epas1*^loxP/loxP^ [C57BL/6JGpt-*Epas1*em1Cflox/Gpt, Strain NO.T008909] strain was purchased from GemPharmatech (Nanjing, Jiangsu, China). *Hif1a* ^loxP/loxP^ strain was a generous gift from the laboratory of Dr. Liwei Xie, Guangdong Academy of Sciences. *Pdgfrb-*CreER^T2^; *Epas1*^loxP/loxP^ (*Epas1*-bKO) mice were generated by breeding *Pdgfrb-*CreER^T2^ transgenic mice to animals carrying floxed *Epas1* (HIF2α protein encoding gene) alleles (*Epas1*^loxP/loxP^). *Pdgfrb-*CreER^T2^; *Hif1a* ^loxP/loxP^ (*Hif1a*-bKO) mice were generated by breeding *Pdgfrb-*CreER^T2^ transgenic mice to animals carrying floxed *Hif1a* alleles (*Hif1a* ^loxP/loxP^). In *Pdgfrb-*CreER^T2^; *Hif1a*-bKO and *Pdgfrb-*CreER^T2^; *Epas1*-bKO mice, *Hif1a* and *Epas1* genetic ablation was induced with daily injection of 100 mg/kg TAM for 5 consecutive days. The deletion of the indicated genes in WAT PDGFRβ + cells were confirmed 7 days following the final TAM injection. In HFD studies, mice were kept on a HFD (60 kcal% fat, Research Diets, D12492i) for the indicated duration of the experiments. For glucose tolerance tests (GTT) and insulin tolerance test (ITT), mice were respectively subjected to an overnight fasting for GTT or 6-h fasting. Animals were then i.p. injected with glucose (Sigma, G7021) at a dosage of 1 g per kg body weight or human insulin at 0.75 U (Sigma, I6634) per kg body weight. Tail vein blood samples were collected at 0, 15, 30, 60, 90, and 120 min post-injection for glucose concentration measurement using glucose meters (Bayer Contour). Mice were maintained with a 12-h light/dark cycle and were provided free access to both food and water. All animals used in this study were on C57BL/6 background.

### Histological analysis

Liver and adipose tissue samples were dissected and rinsed in PBS (Beyotime, #ST448) before subsequent overnight fixation in 4% paraformaldehyde (Beyotime, #P0099). The paraffin embedding, sectioning, and H&E staining were conducted by Wuhan Servicebio Technology Co., Ltd (Wuhan, Hubei, China). Images (bright-field and fluorescence) were captured using an ECHO RVL-100-G microscope. Analysis of adipocyte size utilized bright-field H&E staining images, and the quantification was performed with ImageJ software.

### Tissue and serum measurements

The serum and hepatic levels of triglyceride (TG) in mice were quantified using the TG determination kit (Sigma-Aldrich, T2449&F6428). Hepatic cholesterol (CHO) concentrations were determined using Amplex Red Cholesterol Assay Kit (Invitrogen, #A12216). Serum adiponectin levels were quantified using a mouse adiponectin ELISA (Sigma-Aldrich, EZMADP-60 K). Mouse insulin ELISA kit (Crystal Chem, #90080) was used for the determination of serum insulin levels. TNF-α ELISA kits (BioLegend, #430901) were employed for measuring serum TNF-α levels. All assays were conducted following manufacturer’s instructions.

### Analysis and isolation of WAT PDGFRβ + subpopulations

The analysis and isolation of WAT PDGFRβ + subpopulations followed a previously outlined procedure [27, 36]. Comprehensive experimental procedures have been described in a published protocol [62]. Briefly, white fat depots were minced and incubated in digestion buffer containing (1 × Hank’s Balanced Salted Solution, 1.5% bovine serum albumin and 1 mg/mL Collagenase D (Roche, #11088882001) at 37 °C in a shaking water bath for 1 h. Subsequently, the digested mixture was sequentially filtered through a 100 μm cell strainer, followed by and then a 40 μm cell strainer. The SVF cells underwent a short incubation in 1 mL 1 × RBC lysis buffer (eBioscience, #00–4300–54) to lyse red blood cells and then resuspended in blocking buffer (2% FBS/PBS containing anti-mouse CD16/CD32 and Fc Block at concentration of 1:200). The primary antibodies were then added to the cells in blocking buffer for a 15-min incubation at 4 °C. Subsequently, the cells underwent a single wash and were resuspended in 2% FBS/PBS before sorting. FACS was conducted using a BD Biosciences FACSAria cytometer at the Flow Cytometry Core Facility of the Shanghai Institute of Immunity and Infection.

The primary antibodies and dilutions used in FACS isolation were listed in Supplemental Table 1.

### In vitro adipogenic differentiation and Oil Red O-staining

Isolated primary PDGFRβ + cells were cultured in growth media containing DMEM/F12 (Life Techonologies, #10565042) plus 10% FBS (Sigma, #F8318) until confluency. Confluent cultures were differentiated with adipogenic cock tail as described previously [63]. In vitro differentiated adipocytes were fixed in 10% formalin for 10 min at room temperature for a duration of 10 min. After fixation, the cells underwent 2 washes with deionized water and were then incubated in 60% isopropanol for 5 min. Subsequently, the cells were air-dried completely at room temperature before applying the Oil Red O working solution (2 g/L Oil Red O in 60% isopropanol). After incubating at room temperature for 10 min, the Oil Red O solution was removed, and the cells were washed four times with deionized water before capturing images for analysis. The images were captured for analysis.

### Western blot analysis

Protein extracts were prepared from fresh tissue and cultured cells through mechanical homogenization in NP-40 lysis buffer (Beyotime, #P0013F) supplemented with Protease Inhibitor Cocktail (Sigma, P8340) and Phosphatase Inhibitor Cocktails (Sigma, P0044&P5726). The protein extracts were separated via SDS-PAGE electrophoresis and subsequent transfer onto a PVDF membrane (Millipore, IPFL00010). Following an overnight incubation at 4 °C with the indicated primary antibodies, the blots were exposed to secondary antibodies and visualized using Pierce ECL Plus Western Blotting Substrate (Thermo Fisher Scientific, 32109). The primary antibodies and dilutions used in this study were listed in Supplemental Table 1.

### Quantitative RT-PCR analysis

TRIzol reagent (Invitrogen, 15596018) was used for the isolation of total RNA from fresh tissue and culture cells following the manufacturer’s instructions. First strand cDNA libraries were built using reverse transcription with M-MLV reverse transcriptase (Invitrogen, #28025–021) and random hexamer primers (Invitrogen, #48190011). mRNA relative expression levels were assessed through quantitative PCR (qPCR) using TransStartR Tip Green quantitative PCR SuperMix (TransGen Biotech, #AQ132-21), and *Rps18* levels were used for normalization. qPCR primer sequences employed in this study are provided in Supplemental Table 2.

### Statistical analysis

Statistical analyses were conducted using GraphPad Prism7.0 (GraphPad Software, Inc., La Jolla, CA, USA). A two-tailed unpaired Student’s t-test was employed for comparisons between two independent groups, while two-way ANOVA was utilized for comparisons involving four or more groups. All data were presented as mean ± SEM. No samples or animals were excluded from the analysis. A significance threshold of *P* < 0.05 was set. The anticipated number of independent replicates per group was determined based on previous studies and expertise.

## Results

### Characterization of HIF2α-deficient PDGFRβ + cells isolated from WAT

Evidence has underlined the importance of stromal cell HIF1α signaling in WAT collagen deposition and adipocyte progenitor differentiation, and therefore drives pathologic WAT expansion in diet-induced obese mice [36]. Of note, previous data showed that CRISPR/Cas9-mediated HIF2α inactivation does not affect adipogenic differentiation of isolated PDGFRβ + cells in vitro [36], in contrast to that HIF1α-deficient cells are more resistant to HIF chemical stabilizer-induced inhibition on adipogenesis [36]. Notably, emerging roles of WAT resident PDGFRβ + cells beyond adipogenesis has been documented [64] but were not assessed in the previous work. Moreover, the significance of PDGFRβ + cell HIF2α signaling in WAT remodeling remains undetermined in vivo.

**Fig. 1:**
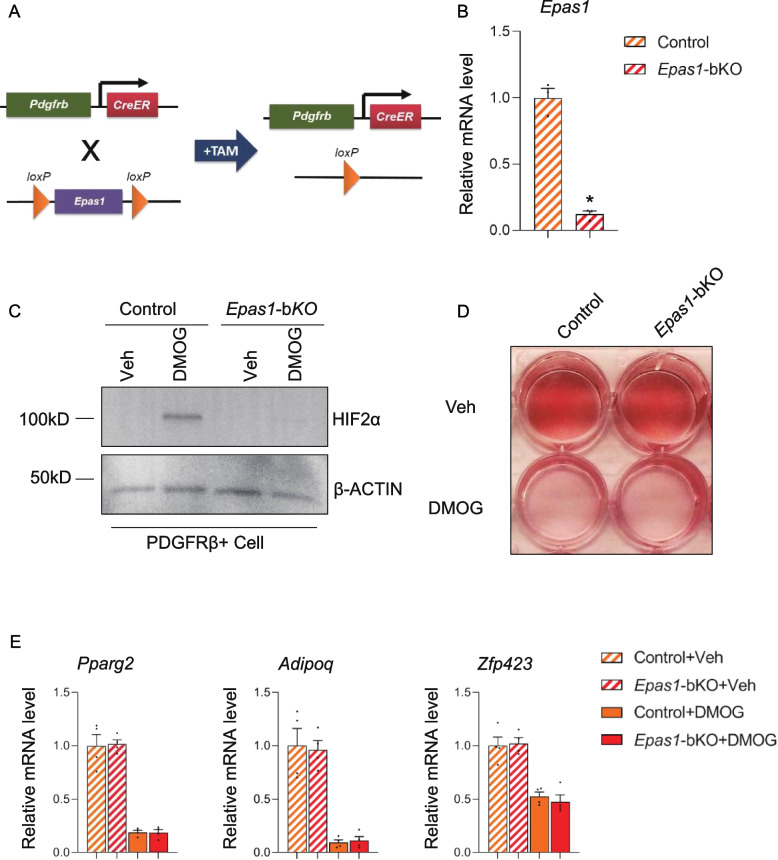
HIF2α inactivation does not impact adipogenic differentiation or inflammatory response in adipose tissue PDGFRβ + cells. **A** Schematic illustration of *Epas1*-bKO mice. *Pdgfrb*-CreERT2; *Epas1*loxP/loxP (*Epas1*-bKO) mice are generated by cross-breeding animals carrying floxed *Espa1* (HIF2α protein encoding gene) alleles (*Epas1*loxP/loxP) to *Pdgfrb*-CreERT2 transgenic mice. *Pdgfrb*-CreERT2 littermates were used as control animals. **B** mRNA levels of *Epas1* in isolated gWAT PDGFRβ + cells from TAM-treated control and *Epas1*-bKO mice. *n* = 3 per group. **C** Western blot analysis of HIF2α and β-ACTIN proteins in isolated gWAT PDGFRβ + cells from TAM-treated control and *Epas1*-bKO mice after the indicated treatments. **D** Representative Oil Red O-staining photos of isolated gWAT PDGFRβ + cells after the induction of in vitro adipogenesis with the indicated treatments. **E** mRNA levels of the indicated mature adipocyte genes in isolated gWAT PDGFRβ + cells after the induction of in vitro adipogenesis with the indicated treatments. *n* = 4 per group

To assess the functional consequences resulting from HIF2α inactivation in PDGFRβ + cells, a new mouse strain was generated, in which *Epas1* (HIF2α encoding gene) is abrogated in *Pdgfrb*-expressing cells in a TAM-inducible manner (*Pdgfrb*-CreER^T2^, *Epas1*^loxP/loxP^; denoted as *Epas1*-bKO mice in this paper) (Fig. 1A). 8-week old *Epas1*-KO and control mice were i.p. injected with TAM (100 mg/kg) for 5 days prior to the isolation of gWAT PDGFRβ + cells using the previously established protocol [27]. The loss of *Epas1* expression in the isolated cells were evaluated by qPCR analysis, and the results showed that the expression level of *Epas1* decreased by ~ 80% in isolated gWAT PDGFRβ + cells (Fig. 1B). In addition, western blot analysis of DMOG (chemical HIF protein stabilizer)-treated cell lysates confirmed HIF2α inactivation in isolated PDGFRβ + cells from this new mouse system. The augmented abundance HIF2α protein induced by DMOG was absent in cultured gWAT PDGFRβ + cells isolated from *Epas1*-bKO mice exposed to TAM (Fig. 1C). The adipogenic potential of PDGFRβ + cells isolated from TAM-treated control and *Epas1*-bKO mice were subsequently assessed. Both control and *Epas1*-deficient WAT PDGFRβ + cells displayed an equivalent degree of in vitro differentiation into adipocytes, as reflected by similar levels of lipid accumulation (Oil Red O staining) (Fig. 1D) and comparable expression levels of mature adipocyte genes (*Pparg2*, *Adipoq* and *Zfp423*) (Fig. 1E). Moreover, both control and HIF2α-deficient PDGFRβ + cells were sensitive to DMOG-induced inhibition on adipogenesis. Consistent with published results [36], the inactivation of *Epas1* in PDGFRβ + cells did not lead to resistance to DMOG-induced inhibition of adipogenic differentiation in vitro as evidenced by lipid accumulation and expression of mature adipocyte genes (Fig. 1D and E). Hence, inactivation of HIF2α does not impact adipogenic differentiation of WAT PDGFRβ + cells in culture.

### PDGFRβ + cell HIF2α inactivation does not result in significant impacts on WAT metabolic remodeling in diet-induced obesity

**Fig. 2:**
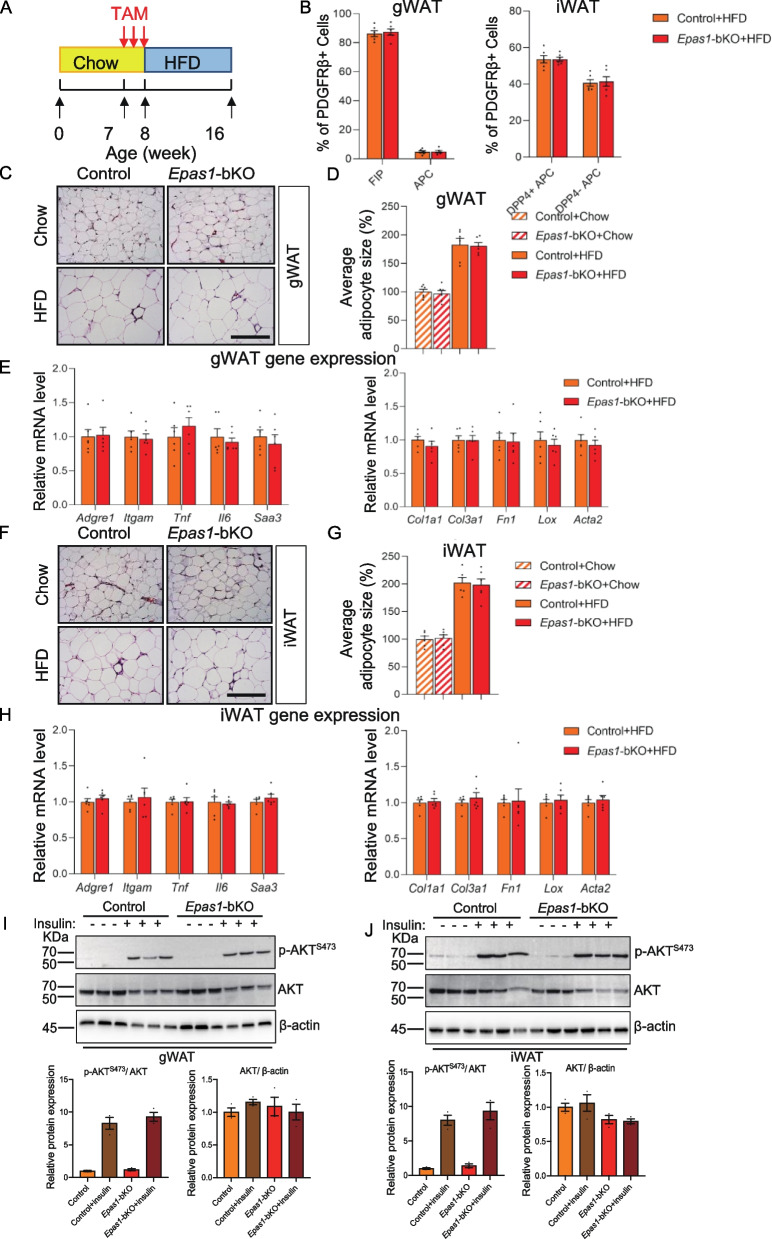
Inducible Hif2α deletion in PDGFRβ + cells does not affect adipose tissue remodeling in diet-induced obesity. **A** Schematic diagram illustrating HFD feeding experiment. Male control or *Epas1*-bKO mice were kept on normal chow diet until 8 weeks of age before being switched to HFD feeding for another 8 weeks. Mice were i.p. injected with TAM (100 mg/kg) for 5 five consecutive days before the diet switch. **B** Relative frequency of PDGFRβ + subpopulations within gWAT and iWAT after 8 weeks of HFD feeding. *n* = 6 per group. **C** Representative gWAT H&E staining images of control and *Epas1*-bKO mice after HFD feeding. Scale bar denotes 200 µM. **D** Average gWAT adipocyte size of control and *Epas1*-bKO mice after HFD feeding. *n* = 6 per group. **E** Inflammation- and fibrosis-related gene expression within gWAT from control and *Epas1*-bKO mice after HFD feeding. *n* = 6 per group. **F** Representative iWAT H&E staining images of control and *Epas1*-bKO mice after HFD feeding. Scale bar denotes 200 µM. **G** Average iWAT adipocyte size of control and *Epas1*-bKO mice after HFD feeding. *n* = 6 per group. **H** Inflammation- and fibrosis-related gene expression within iWAT from control and *Epas1*-bKO mice after HFD feeding. *n* = 6 per group. **I** Western blot analysis of phosphorylated AKT (pAKT), total AKT and β-actin in gWAT whole tissue extracts from control and *Epas1*-bKO mice after HFD feeding. For quantification, intensity of pAKT band is normalized to that of total AKT band, and intensity of total AKT is normalized to that of β-actin. **J** Western blot analysis of phosphorylated AKT (pAKT), total AKT and β-actin in iWAT whole tissue extracts from control and *Epas1*-bKO mice after HFD feeding. For quantification, intensity of pAKT band is normalized to that of total AKT band, and intensity of total AKT is normalized to that of β-actin

Previously published scRNA-seq analysis identified molecularly and functionally distinct culsters of PDGFRβ + mural cells in gWAT and iWAT [27, 36, 38]. Within gWAT, LY6C + PDGFRβ + cells represent FIPs which are enrich with inflammation- and fibrosis-related gene signature. FIPs lack adipogenic potential, and even exhibit inhibitory effects on de novo adipogenesis, while LY6C- PDGFRβ + APCs are highly adipogenic [27, 37, 38]. Two PDGFRβ + subpopulations on the basis of Dpp4 expression were separated within iWAT [36]. Both iWAT DPP4 + and DPP4– PDGFRβ + cells can differentiate into mature adipocytes in vitro and in vivo [34, 36]. Thus, within iWAT, there exits two PDGFRβ + APC subgroups, denoted as DPP4 + and DPP4- APCs. In multiple strains of mouse models, alterations in the frequency of PDGFRβ + cell subpopulations have been linked to adipose tissue remodeling during diet-induced obesity, serving as an indicator of metabolic status of WAT [36, 37, 39]. Suppression of pathological activation of HIF1α signaling has been shown to increase the relative frequencies of adipogenic gWAT APCs and iWAT DPP4 + APCs, which is associated with enhanced protective adipocyte hyperplasia in obesity [36]. Hence, to determine whether HIF2α inactivation affects the relative frequency of these PDGFRβ + cell subpopulations across depots, 8 weeks old Epas1-bKO and control mice were first i.p. injected with TAM (100 mg/kg) for 5 consecutive days to induce Epas1 inactivation, before being switched to HFD for another 8 weeks (Fig. 2A). At the conclusion of HFD feeding, the relative abundance of PDGFRb + cell subpopulations in both gWAT and iWAT was quantified by FACS analysis. Compared to those in control mice, HIF2α deletion in PDGFRβ + cells did not lead to differences in the frequency of FIPs and APCs in gWAT, or DPP4 + and DPP4- APCs in iWAT after 8 weeks of HFD feeding (Fig. 2B). As abovementioned, HIF1α has been documented as critical regulator in PDGFRb + cells within WAT [36]. HIF1α-triggered PDGFRβ signaling within WAT PDGFRβ + cells stimulates inhibitory serine 112 phosphorylation of PPARγ, subsequently blocking adipogenesis. Suppression of HIF1α in PDGFRβ + progenitors promotes adipogenesis within subcutaneous and intra-abdominal depots, leading to healthy WAT remodeling and improved metabolic health in obesity. These protective effects are recapitulated by treating obese mice with the PDGFR antagonist Imatinib, which enhances adipocyte hyperplasia and glucose tolerance in a manner dependent on progenitor cell PPARγ.

Whether the ablation of HIF2α in PDGFRβ + cells leads to phenotypical changes at the tissue level were subsequently evaluated in the HFD-induced obese control and *Epas1*-bKO mice (Fig. 2A). Histological analysis based on the H&E staining of WAT sections from HFD-fed *Epas1*-bKO and control mice showed no significant morphological changes in gonadal or inguinal WAT depots (Fig. 2C and F), and no difference in average sizes of fat cells was detected (Fig. 2D and G). Furthermore, gene expression analysis did not reveal different expression of inflammation (*Adgre1*, *Itgam*, *Tnf*, *Il6*, *Saa3*)—or fibrosis (*Col1a*, *Col3a1*, *Fn1*, *Lox*, *Acta2*) -related genes in gWAT and iWAT from *Epas1*-bKO and control mice after HFD feeding (Fig. 2E and H). In addition, levels of insulin-induced pAKT in both gWAT and iWAT of these mice were equivalent to those observed in corresponding tissues from control animals, reflective of similar degree of WAT insulin sensitivity (Fig. 2I and J). Collectively, these results revealed that *Epas1*-bKO mice did not exhibit a local phenotype within WAT depots in the context of obesogenic diet-induced WAT expansion.

### No apparent changes of hepatic lipid accumulation or systemic metabolic homeostasis in HFD-fed *Epas1*-bKO mice

As above mentioned, accumulating evidence validates that the manner in which WAT undergoes remodeling substantially influences liver energy balance and systemic metabolic health in obesity [14, 23]. For example, in the previous study, the mouse model in which the expression of a dominant-negative form of HIF1α is induced in *Pdgfrb*-expressing cells to suppress HIF1α was employed to promote healthy adipose tissue remodeling in obese mice [36]. HIF1α suppression in PDGFRβ + cells promotes adipocyte hyperplasia in intra-abdominal and subcutaneous depots, and healthy WAT remodeling in obese mice, associated with remarkable reduction of hepatic lipid accumulation [36]. However, a caveat to the dominant-negative form is that it can inhibit the activity of both HIF1α and HIF2α in principle [65]. To confirm the effects of PDGFRβ + cell HIF1α inactivation on WAT remodeling and liver lipid accumulation in obesity, a new TAM-inducible *Hif1a* knockout system (*Pdgfrb*-CreER^T2^, *Hif1a*^loxP/loxP^; herein denoted as *Hif1a*-bKO mice) were generated (Fig. S2A). 8 weeks old *Hif1a*-bKO and control mice were i.p. injected with TAM (100 mg/kg) for 5 consecutive days, followed by an additional 8 weeks of HFD feeding (Fig. S2B). The loss of *Hif1a* expression was evaluated in isolated PDGFRβ + cells 1 week after TAM administration. qPCR analysis showed ~ 75% reduction of *Hif1a* mRNA levels in PDGFRβ + cells isolated from *Hif1a*-bKO mice following TAM treatment (Fig. S2C). Phenotypically, no body weight difference between control and *Hif1a*-bKO mice fed on both chow and HFD diets were observed during HFD feeding (Fig. S2D). The GTT showed improvement of glucose metabolism in HFD-fed *Hif1a*-bKO mice (Fig. S2F), in agreement with the above-mentioned model of PDGFRβ + cell HIF1α loss-of-function model used in the previous study [36]. Of note, the liver lipid accumulation was remarkably reduced by ~ 25% in obese *Hif1a*-bKO mice after 8 weeks HFD feeding (Fig. S2G and H), highlighting the protective effects on the development of fatty liver disease via HIF1α suppression in PDGFRβ + cells.

**Fig. 3:**
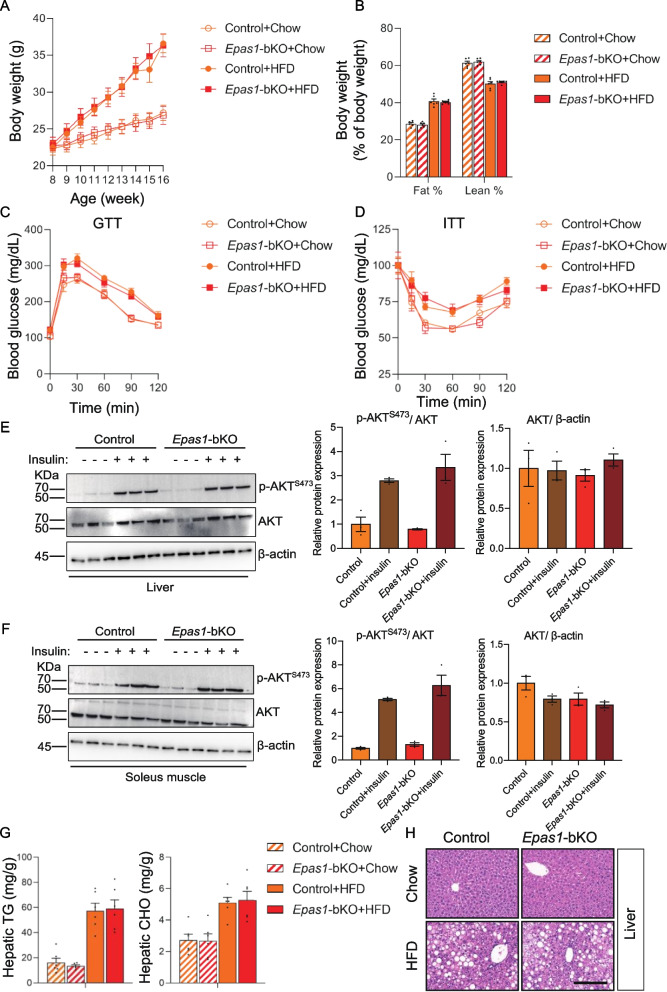
No apparent systemic effects caused by PDGFRβ + cell HIF2α inactivation in diet-induced obesity. **A** Monitoring of weekly body weights in control and *Epas1*-bKO mice during 8 weeks of HFD feeding. *n* = 6 per group. **B** Body composition (% of fat mass and lean mass) of control and *Epas1*-bKO mice after HFD feeding. *n* = 6 per group. **C** Glucose tolerance test (GTT) of HFD-fed control and *Epas1*-bKO mice. *n* = 6 per group. **D** Insulin tolerance test of (ITT) of HFD-fed control and -bKO mice. *n* = 6 per group. **E** Western blot analysis of phosphorylated AKT (pAKT), total AKT and β-actin in liver extracts from control and *Epas1*-bKO mice after HFD feeding. For quantification, intensity of pAKT band is normalized to that of total AKT band, and intensity of total AKT is normalized to that of β-actin. **F** Western blot analysis of phosphorylated AKT (pAKT), total AKT and β-actin in soleus muscle extracts from control and *Epas1*-bKO mice after 8 weeks of HFD feeding. For quantification, intensity of pAKT band is normalized to that of total AKT band, and intensity of total AKT is normalized to that of β-actin. **G** Hepatic TG and CHO contents of control and *Epas1*-bKO mice after 8 weeks of HFD feeding. *n* = 6 per group. **H** Representative liver H&E staining images of HFD-fed control and *Epas1*-bKO mice. Scale bar denotes 200 µM

Hence, we next set to determine whether PDGFRβ + cell HIF2α inactivation would be associated with changes of hepatic fat content and systemic metabolism in the setting of obesity. Similar to the findings in *Hif1a*-bKO mice, no significant difference of body weight and body composition were found between control and *Epas1*-bKO mice subjected to HFD feeding (Fig. 3A and B). In line with the lack of phenotypic changes in *Epas1*-bKO WAT depots, PDGFRβ + cell HIF2α had no effect on circulating parameters closely linked to the metabolic health status of WAT or systemic energy homeostasis (Table 1). More specifically, serum levels of metabolically beneficial adiponectin were not changed in HFD- and chow- fed *Epas1*-bKO mice (Table 1). Similarly, the levels of circulating TNF-α, a major pro-inflammatory factor reflecting the health status of adipose tissue and systemic metabolism, remained unchanged in obese *Epas1*-bKO mice (Table 1). With respect to lipid metabolism, there were same serum TG concentrations observed between the two genotypes (Table 1). The GTT and ITT were conducted on both chow- and HFD-fed control and *Epas1*-bKO mice to evaluate the impacts on systemic glucose metabolism. However, no significant difference was detected (Fig. 3C and D). These results were in alignment with unchanged levels fasting glucose and serum insulin in chow-fed lean and HFD-fed obese *Epas1*-bKO mice (Table 1). The lack of impact of PDGFRβ + cell HIF2α inactivation on glucose metabolism was further supported by equivalent levels of insulin action, reflected by the same degree of insulin-induced AKT phosphorylation, in non-adipose peripheral tissues including liver and soleus muscle (Fig. 3E and F). Consistently, unlike the results observed in PDGFRβ + cell HIF1α inactivation model (Fig. S2G and H), no significant difference in hepatic lipid accumulation was detected in HFD-fed *Epas1*-bKO mice (Fig. 3G and H). These findings underscore the distinct functional roles of HIF isoforms in this specific context. In conclusion, metabolic phenotyping of *Epas1*-bKO did not uncover remarkable effects on hepatic or systemic metabolism caused by HIF2α inactivation in PDGFRβ + mural cells.

**Table 1:** Serum measurements of *Epas1*-bKO mice after 8 weeks of HFD feeding

	Control + Chow	*Epas1*-bKO + Chow	Control + HFD	*Epas1*-bKO + HFD
Serum TG (mg/dL)	47.73 ± 3.7	44.56 ± 3.3	93.14 ± 5.1^*^	96.81 ± 4.6^#^
Serum Adiponectin (μg/mL)	9.16 ± 0.5	9.89 ± 0.9	5.98 ± 0.2	5.9 ± 0.4
Serum TNF-α (pg/mL)	18.42 ± 2.1	20.83 ± 3.2	38.84 ± 3.5^*^	40.49 ± 4.4
Fasting glucose (mg/dL)	96.83 ± 7.2	100.5 ± 7.3	145.17 ± 7.9^*^	139.33 ± 13.1^#^
Fasting insulin (ng/mL)	0.53 ± 0.1	0.51 ± 0.1	0.9 ± 0.1	0.93 ± 0.1

Values are mean ± SEM (*n* = 6 per group). ^*^P < 0.05 control mice compared with HFD mice by two-way ANOVA. ^#^*P* < 0.05 *Epas1*-bKO mice fed with chow diet compared with *Epas1*-bKO mice fed with HFD by two-way ANOVA

## Discussion

HIFα proteins were first described as transcription factors mediating oxygen-dependent responses which modulate a variety of cellular processes [66]. Despite of the less established HIF3α protein, HIF1α and HIF2α isoforms are documented in the literature as important regulators of WAT function, in particular in the setting of calorie overload [40]. In WAT, HIF1α functions in mature adipocytes, macrophages and stromal cells to regulate fibrogenic and pro-inflammatory gene programs [36, 48–50, 57], while HIF2 α has been indicated to be protective for WAT metabolic health [54–56, 60]. In particular, recent findings revealed the significant role for HIF1α signaling in controlling gene expression associated with fibrogenesis and adipogenesis in adipose stromal cells, and thus impact the metabolic health of WAT in obesity [36]. In addition to its regulation of fibrogenic gene expression, HIF1α signaling exerts substantial effects on adipocyte progenitor differentiation in the settings of HFD-induced white adipocyte hyperplasia and cold-induced beige adipogenesis [67]. HIF1α activation suppresses PPARg activity, in part, through its downstream signaling events which lead to inhibitory phosphorylation of PPARg at serine 112 [36]. Therefore, suppression of HIF1α activity in PDGFRβ + cells facilitates healthy WAT expansion via downregulation of tissue fibrosis, local inflammation, and enhance recruitment of functional new adipocytes [36]. These effects on WAT remodeling are associated with metabolic benefit in non-adipose tissue including improved hepatic lipid metabolism [36], highlighting the importance of WAT mesenchymal stromal cells in systemic metabolic health [64].

Unlike the notable phenotype in PDGFRβ + cell HIF1α-deficient mice (Fig. S2F, G and H), the data in this study do not uncover significant changes regarding the function of PDGFRβ + cells, WAT metabolic health, or liver fat content when HIF2α is inactivated in PDGFRβ + cells. TAM-inducible deletion of *Epas1* gene in PDGFRβ + cells during HFD feeding does not affect the inflammation response in WAT, nor does it influence adipogenic differentiation of PDGFRβ + cell in culture. The characterization of WAT from HFD-fed mice fails to distinguish *Epas1*-bKO mice from wild type controls. In alignment, no significant changes of key metabolic parameters reflective of systemic metabolic status are recorded in *Epas1*-bKO mice fed with HFD.

These results further corroborate the existing evidence showing the functional distinction between HIF isoforms in a number of settings. For example, both HIF1α and HIF2α proteins have been extensively studied in cancer cells [42, 68]. Increased levels of HIF1α and HIF2α have been associated with poor prognosis in various human cancer types [42]. Of note, although the two HIFα proteins have initially showed to have much overlapping functions, growing evidence has revealed that HIF1α and HIF2α can have dichotomous effects in the same cell type. It is now clear that HIF1α and HIF2α independently regulate the expression of distinct target genes, despite of sharing a larger number of common target genes involved in cellular hypoxic responses [42, 69, 70]. In terms of metabolism and metabolic disease, the role of HIF1α has been well-established in the regulation of sterile inflammation, tissue fibrosis, insulin action, and lipid handling in various peripheral metabolic organs/tissues including liver, intestine, pancreatic β-cell, and adipose tissue [40]. Considering the strong evidence regarding the critical roles of HIF proteins in energy metabolism, HIF inhibitors are currently undergoing pre-clinical and clinical evaluations as potential therapeutics for human diseases [40, 60, 71]. Therefore, the thorough understanding of unique properties of HIFα isoforms is warranted to identify new therapeutic opportunities addressing metabolic conditions encompass the liver, including nonalcoholic fatty liver disease. In conjunction with prior pertinent studies [36, 48–50, 52, 54–61], these results have implicated that HIF2α, unlike HIF1α, is not essential in PDGFRβ + cells to maintain WAT and systemic metabolic homeostasis in HFD-fed mice, suggesting that selective pharmacological targeting of HIF1α rather than HIF2α may represent an effective strategy to promote WAT metabolic health, and preserve hepatic and systemic energy balance in the context of obesity.

### Study strengths and limitations

This study emphasizes the significance of the isoform-specific roles of HIFα proteins in the regulation of adipose tissue biology. The robust methodology, incorporating genetic models and metabolic phenotyping, enhances the credibility of the findings. This study poses limitations represented by that the potential involvement of *Epas1* inactivation within liver PDGFRβ + cells cannot be ruled out as a factor in the control of hepatic steatosis, a caveat to the mouse models. However, the absence of evident phenotypic changes in *Epas1*-bKO mice contradicts the functional significance of HIF2α in PDGFRβ + cells localized either in liver or WAT. Furthermore, emerging evidence indicates that the regulatory mechanisms governing adipocyte progenitors exhibit significant sex-dependent differences [39, 72]. Hence, another limitation of this study lies in its exclusive focus on male animals when investigating the functions of HIF isoforms. Future research should encompass female subjects to explore potential sex-dependent differences in the roles of HIF isoforms in WAT mesenchymal cell populations, thereby providing a more comprehensive understanding. Lastly, the time-dependent activation of different HIF proteins might be another factor contributing to their distinct functions. The chronic activation pattern of HIF2α, compared to the more acute activation of HIF1α, might represent another possible mechanism governing the isoform-dependent actions of HIF proteins, which warrants investigation in future studies.

## Conclusions

Healthy WAT expansion plays a crucial role in maintaining liver energy balance, mitigating obesity-related hepatic steatosis. An essential feature of metabolically healthy WAT is the increased recruitment of adipocytes originating from adipose precursor cells, which confers protective effects to the development of liver steatosis in obesity. This study suggests that the inducible inactivation of HIF2α in PDGFRβ + cells does not yield noticeable effects on WAT expansion in obese mice. The adipogenic capacity of PDGFRβ + cells remains largely unaltered following genetic HIF2α ablation. Furthermore, there is no differences in key parameters associated with healthy WAT remodeling, including liver fat accumulation in PDGFRβ + cell *Epas1*-deficient obese mice. These findings suggest that, unlike HIF1α, PDGFRβ + cell HIF2α appears to be dispensable for WAT metabolic remodeling and its downstream effects on liver metabolic homeostasis in diet-induced obesity. Overall, this study highlights the distinct roles of HIF protein isoforms in the regulation of adipose tissue biology, providing new insights for the development of clinical drugs targeting obesity-related hepatic steatosis.

### Supplementary Information

**Supplementary material 1.**

## Data Availability

No datasets were generated or analysed during the current study.

## Abbreviations

NAFLD: Nonalcoholic fatty liver disease
NASH: Nonalcoholic steatohepatitis
APCs: Adipocyte precursor cells
FIPs: Fibro-inflammatory progenitors
gWAT: Gonadal white adipose tissue
iWAT: Inguinal white adipose tissue
HFD: High-saturated fat diet
GTT: Glucose tolerance test
ITT: Insulin tolerance test
H&E: Hematoxylin and eosin
mRNA: Messenger RNA
PCR: Polymerase chain reaction
rtTA: Reverse tetracycline–controlled transactivator
TAM: Tamoxifen
TG: Triglyceride
CHO: Cholesterol
IL: Interleukin
TNF-α: Tumor necrosis factor alpha
WAT: White adipose tissue
WT: Wild-type
SEM: Standard error of the mean
