# Supplementary material for: PDGFRβ + cell HIF2α is dispensable for white adipose tissue metabolic remodeling and hepatic lipid accumulation in obese mice
Source: Lipids Health Dis. 2024 Mar 20;23:81. doi: 10.1186/s12944-024-02069-1 (PMC10953078; doi:10.1186/s12944-024-02069-1)
Supplement: Supplementary file 1 — Supplementary material 1. [file 12944_2024_2069_MOESM1_ESM.docx]

**Supplementary Material**

Table S1: The primary antibodies and dilutions used in this study

| Antibodies | Source | Identifier | Additional information |
| --- | --- | --- | --- |
| CD45-PerCP/Cyanine5.5 | Biolegend | clone 30-F11; Cat# 103132 | 1:400 |
| CD31-PerCP/Cyanine 5.5 | Biolegend | clone 390; Cat# 102420 | 1:400 |
| PDGFRβ-PE | Biolegend | clone APB5; Cat# 136006 | 1:75 |
| LY6C-APC | Biolegend | clone HK1.4; Cat# 128016 | 1:400 |
| CD9-BV421 | BD Biosciences | clone KMC8; Cat# 564235 | 1:250 |
| DPP4-APC | Biolegend | clone H194-112; Cat# 137807 | 1:200 |
| Phospho-AKT (Ser473) | Cell Signaling Technology | Cat# 9271 | 1:1000 |
| AKT | Cell Signaling Technology | Cat #2920 | 1:1000 |
| β-ACTIN | Sigma | Cat #A1978 | 1:10000 |
| HIF2α | Novus | Cat #NB100-122 | 1:100 |
| HIF1α | Cell Signaling Technology | Cat #36169 | 1:1000 |

Table S2: Sequences of qPCR primers used in this study

| No. | Mouse gene | Forward 5’-3’ | Reverse 5’-3’ |
| --- | --- | --- | --- |
| 1 | *Epas1* | GTGACCCAAGACGGTGACAT | TCCCAAAACCAGAGCCGTTT |
| 2 | *Pparg2* | GCATGGTGCCTTCGCTGA | TGGCATCTCTGTGTCAACCATG |
| 3 | *Adipoq* | AGATGGCACTCCTGGAGAGAA | TTCTCCAGGCTCTCCTTTCCT |
| 4 | *Zfp423* | CAGGCCCACAAGAAGAACAAG | GTATCCTCGCAGTAGTCGCACA |
| 5 | *Adgre1* | TTGTACGTGCAACTCAGGACT | GATCCCAGAGTGTTGATGCAA |
| 6 | *Itgam* | ATGGACGCTGATGGCAATACC | TCCCCATTCACGTCTCCCA |
| 7 | *Tnf* | CCTGTAGCCCACGTCGTAG | GGGAGTAGACAAGGTACAACCC |
| 8 | *II6* | AAGCCAGAGTCCTTCAGAGAGA | ACTCCTTCTGTGACTCCAGCTT |
| 9 | *Saa3* | TGCCATCATTCTTTGCATCTTGA | CCGTGAACTTCTGAACAGCCT |
| 10 | *Col1a1* | AGATGATGGGGAAGCTGGCAA | AAGCCTCGGTGTCCCTTCATT |
| 11 | *Col3a1* | ATTCTGCCACCCCGAACTCAA | ACAGTCATGGGGCTGGCATTT |
| 12 | *Fn1* | GAGAGCACACCCGTTTTCATC | GGGTCCACATGATGGTGACTT |
| 13 | *Lox* | TCGCTACACAGGACATCATGC | ATGTCCAAACACCAGGTACGG |
| 14 | *Acta2* | TGACGCTGAAGTATCCGATAGA | GTACGTCCAGAGGCATAGAGG |
| 15 | *Rsp18* | CATGCAAACCCACGACAGTA | CCTCACGCAGCTTGTTGTCTA |


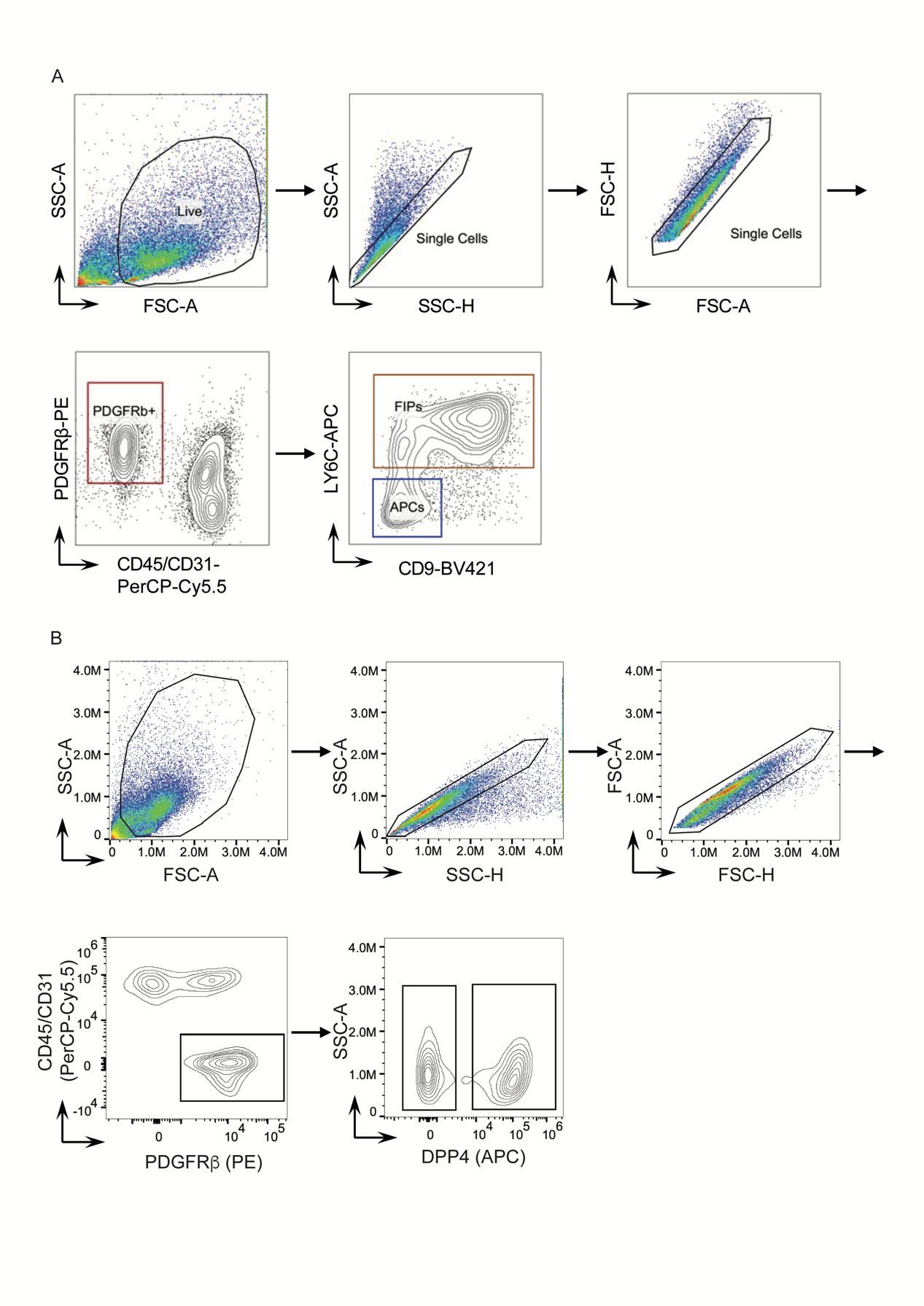


**Fig. S1** FACS collection gates for the isolation of WAT PDGFRβ+ cell subpopulations.

1. FACS strategy for the isolation of mouse visceral WAT (gonadal WAT) PDGFRβ+ subpopulation cells (FIPs and APCs).
2. FACS strategy for the isolation of mouse subcutaneous WAT (inguinal WAT) PDGFRβ+ subpopulation cells (DPP4+ APCs and DPP4- APCs).


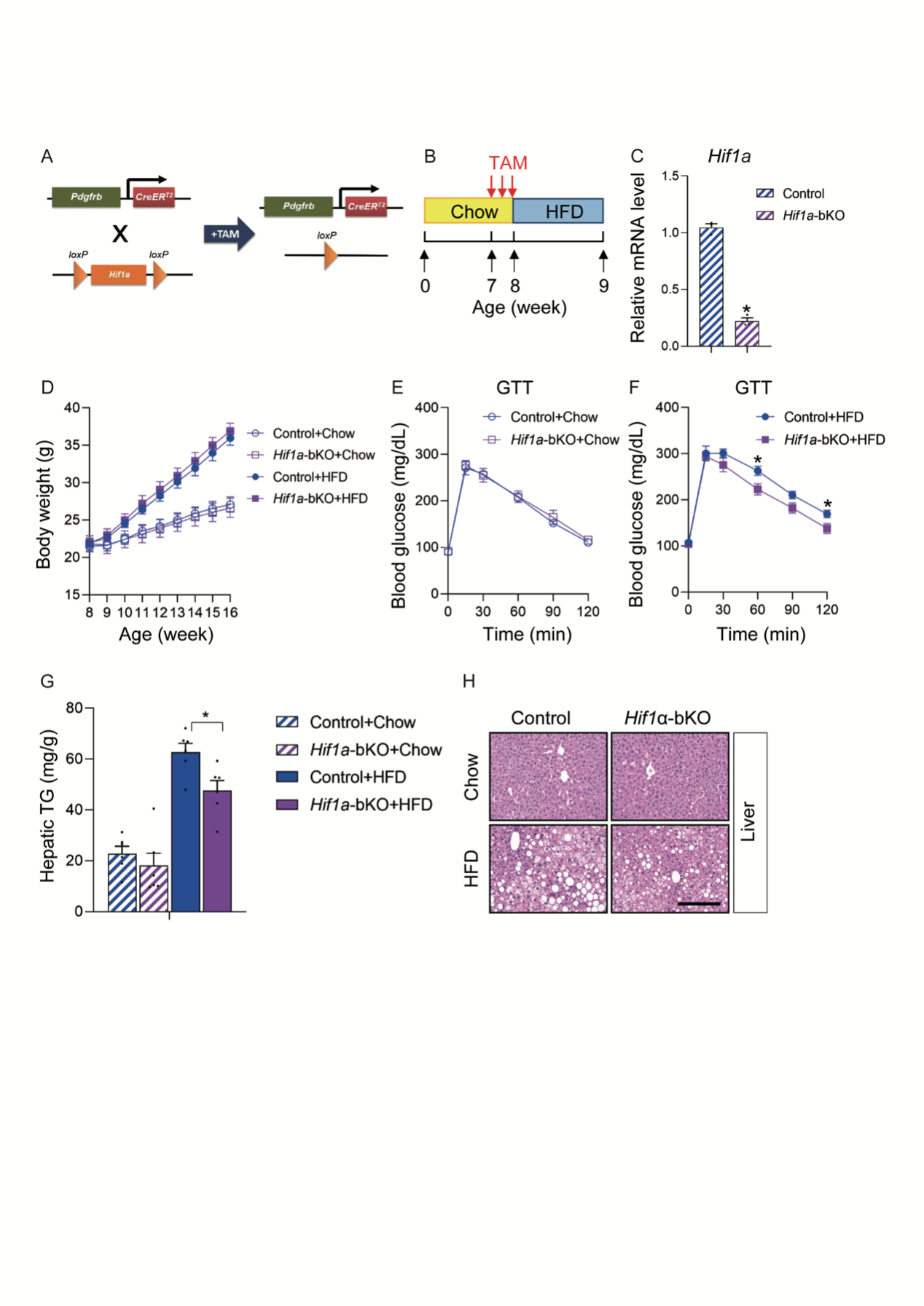


**Fig. S2** PDGFRβ+ cell HIF1α inactivation improves hepatic triglycerides accumulation in diet-induced obesity.

1. Schematic illustration of *Hif1a*-bKO mice. *Pdgfrb*-CreER^T2^; *Hif1a*^loxP/loxP^ (Hif1a-bKO) mice are generated by breeding Pdgfrb-CreER^T2^ transgenic mice to animals carrying floxed *Hif1a* alleles (*Hif1a^l^*^oxP/loxP^). Littermates carrying only *Pdgfrb*-CreER^T2^ were used as control animals.
2. Schematic diagram illustrating HFD feeding experiment. Male control or *Hif1*a-bKO mice were kept on standard chow diet until 8 weeks of age before switched to HFD feeding for another 8 weeks. Mice were i.p. injected with TAM (100 mg/kg) for 5 five consecutive days before the diet switch.
3. mRNA levels of *Hif1a* in isolated gWAT PDGFRb+ cells from TAM-treated control and *Hif1a-*bKO mice. n=3 per group.
4. Weekly body weight of control and *Hif1*a-bKO mice during HFD feeding. n=6 per group.
5. Glucose tolerance tests of control and *Hif1*a-bKO mice fed on chow diet. n=6 per group.
6. Glucose tolerance tests of control and *Hif1*a-bKO mice fed on HFD. n=6 per group.
7. Hepatic triglycerides (TG) contents of control and *Hif1*a-bKO mice after HFD feeding. n=6 per group.
8. Representative H&E staining of liver sections from control and *Hif1*a-bKO mice after HFD feeding. Scale bar denotes 200μM.
